# The two tempos of nuclear pore complex evolution: highly adapting proteins in an ancient frozen structure

**DOI:** 10.1186/gb-2005-6-10-r85

**Published:** 2005-09-30

**Authors:** Eric Bapteste, Robert L Charlebois, Dave MacLeod, Céline Brochier

**Affiliations:** 1 Canadian Institute for Advanced Research Program in Evolutionary Biology, Department of Biochemistry and Molecular Biology, Dalhousie University, College Street, Halifax, Nova Scotia, B3H 1X5 Canada; 2 Genome Atlantic, Department of Biochemistry and Molecular Biology, Dalhousie University, 5850 College Street, Halifax, Nova Scotia, B3H 1X5, Canada; 3 EA EGEE (Evolution, Génome, Environnement), Centre Saint-Charles, Université Aix-Marseille I, place Victor Hugo, 13331 Marseille Cedex 3, France

## Abstract

**Background:**

The origin of the nuclear compartment has been extensively debated, leading to several alternative views on the evolution of the eukaryotic nucleus. Until recently, too little phylogenetic information was available to address this issue by using multiple characters for many lineages.

**Results:**

We analyzed 65 proteins integral to or associated with the nuclear pore complex (NPC), including all the identified nucleoporins, the components of their anchoring system and some of their main partners. We used reconstruction of ancestral sequences of these proteins to expand the detection of homologs, and showed that the majority of them, present all over the nuclear pore structure, share homologs in all extant eukaryotic lineages. The anchoring system, by contrast, is analogous between the different eukaryotic lineages and is thus a relatively recent innovation. We also showed the existence of high heterogeneity of evolutionary rates between these proteins, as well as between and within lineages. We show that the ubiquitous genes of the nuclear pore structure are not strongly conserved at the sequence level, and that only their domains are relatively well preserved.

**Conclusion:**

We propose that an NPC very similar to the extant one was already present in at least the last common ancestor of all extant eukaryotes and it would not have undergone major changes since its early origin. Importantly, we observe that sequences and structures obey two very different tempos of evolution. We suggest that, despite strong constraints that froze the structural evolution of the nuclear pore, the NPC is still highly adaptive, modern, and flexible at the sequence level.

## Abstract 2

An analysis of the taxonomic distribution, evolutionary rates and phylogenies of 65 proteins related to the nuclear pore complex shows high heterogeneity of evolutionary rates between these proteins.

## Background

In 1938, Copeland proposed to gather in a large but unnamed natural group all the organisms (both multicellular and unicellular) harboring a nucleus [1,2]. He considered that the nucleus was too complex a structure to have appeared independently several times [1,2]. The possession of a nucleus is still commonly considered as a good synapomorphy for eukaryotes. However, very little broad comparative analyses of eukaryotic nuclei have been conducted in order to test the homology of this structure. Very recently, Mans *et al. *[3] investigated by BLAST searches the distribution of homologous proteins of the nucleus and of a few associated systems in the three domains of life. Yet, apart from this stimulating work, the nucleus is only well studied in vertebrates [4,5] and in fungi [6-8], whereas little is known in protists or plants. For this reason, the origin and evolution of this structure are difficult to address and largely remain to be described.

The nuclear pore complex (NPC) is one of the most important components of the nucleus. It is a gate between the nucleoplasm and the cytoplasm, mediating the nucleocytoplasmic transport of small molecules by either diffusion or active transport of large substrates [9-15]. Recent works have suggested that some components of the NPC may play a role in the structural and functional organization of perinuclear chromatin [16], in chromatin boundary activities [17] and in interactions with kinetochores [18,19]. A role in numerous pathways has also been observed, such as the control of gene expression, oncogenesis and the progression of the cell cycle [20-23]. The NPC is thus a fully integrated structure and its evolution is likely very constrained.

**Figure 1:**
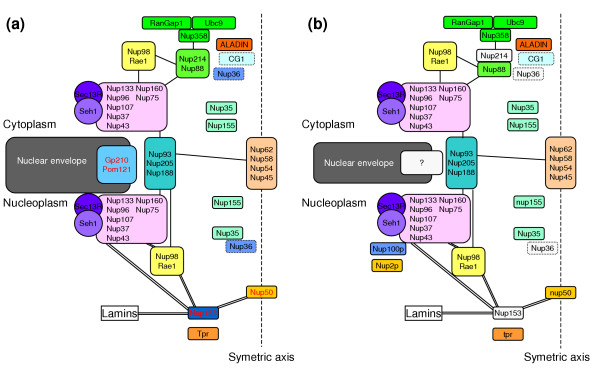
The structure of the nuclear pore complex. Schematic representation of the position of the major nucleoporin subcomplexes in **(a) **unikonts and **(b) **bikonts. The schematic organization of the NPC in unikonts is based on the schematic organizations of NPC in vertebrates published by Powers and Dasso [15], completed accordingly with recent works [5,19]. Boxes delimited by dashed lines indicate proteins having unkown or no precise localization within or around the NPC. Light gray boxes represent nucleoporins present in unikonts but having no homologs in bikonts. Protein names in black in (a) indicate proteins having homologs in fungi, whereas those in red indicate proteins having no homologs but structural analogues in fungi. Lines between subcomplexes indicate putative interactions whereas double lines indicate undisputable interactions.

**Figure 2:**
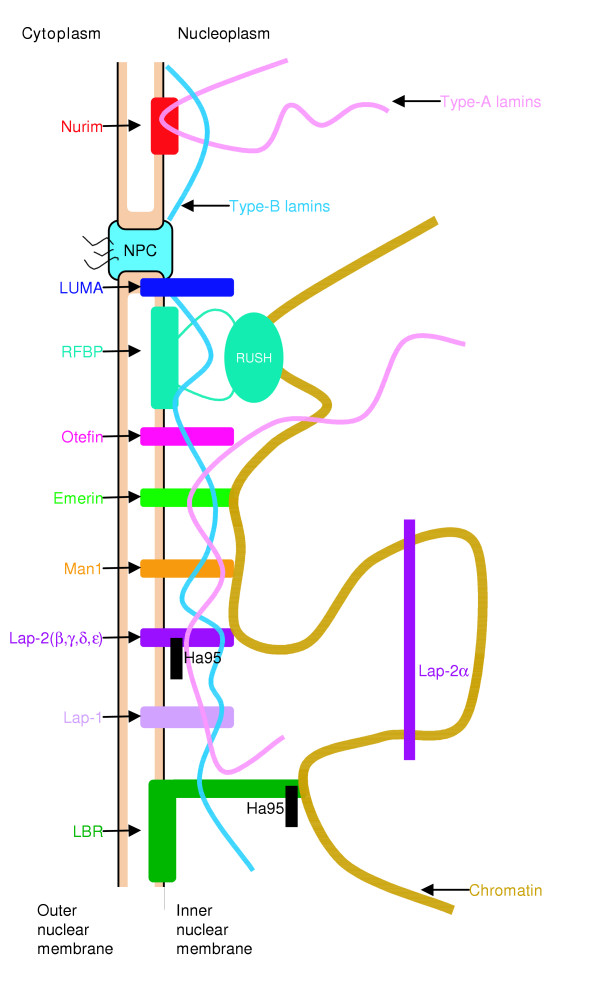
Schematic representation of the putative inner nucleus membrane organization. All the proteins (Nurim, Emerin, Lap-1, Lap-2, A-type lamins and B-type lamins) except Lbr are found only in metazoa (for more details, see [65]). Distant homologs of rfbp and Man1 have been found in some bikont protists (Table 1).

The NPC is also one of the largest macromolecular complexes in the eukaryotic cell (approximately 60 MDa and 125 MDa in yeast [6] and vertebrates [24], respectively), composed of more than 30 different interacting proteins generally referred to as nucleoporins [5,6,15,25]. The nuclear pore exhibits an octagonal symmetry around its cylindrical axis. It consists of a cylindrical core, composed of eight interconnected spokes (each spoke being composed of the Nup93, Nup205, Nup188 nucleoporins; Figure 1a), that surrounds the central channel. Each spoke is connected on the nucleoplasm and cytoplasm sides to a Nup160 subcomplex (Nup133, Nup96, Nup107, Nup37, Nup43, Nup160, Nup75) that binds to the Sec13R and Seh1 proteins (Figure 1a; Table 1). The Nup160 complexes form a plane pseudo-mirror symmetry running parallel to the nuclear envelope. From the central ring, 50 to 100 nm fibrils extend into the nucleoplasm, where they conjoin distally to form a basket-like structure (Nup153, Nup98/Rae1, Nup50, Tpr; Figure 1a; Table 1), spreading outwards into the cytoplasm (Nup214, Nup88, Nup358, Ubc9, RanGap1, Nup35; Figure 1a; Table 1). The Nup62 subcomplex, also called the central transporter, may be involved in transport across the NPC (Figure 1a; Table 1). In vertebrates, the NPC is anchored to the nuclear envelope by the Gp210 and the Pomp121 proteins (Figure 1a) and is connected with the nuclear lamina, a meshwork of lamins and lamin-associated proteins that form a 15 nm thick fibrous structure between the inner nuclear membrane and peripheral chromatin (Figure 2).

To further highlight the origin and the evolution of this essential structure in eukaryotes, we investigated the evolutionary history of its components using a classic phylogenetic approach. Beyond detection of homologs by BLAST, we studied the phylogenies, the evolutionary rates, and the domain organization of all the known nucleoporins and of a selection of their main partners involved in nuclear transport or composing the nuclear envelope. We subsequently propose some hypotheses on the origin of the nucleus and its evolution.

## Results and discussion

### Identification of the core of homologous NPC and NPCa proteins present in all extant eukaryotes

Our first goal was to test the widely but *a priori *accepted hypothesis that the NPC is homologous in all extant eukaryotes by investigating the distribution of homologs of the metazoan NPC and NPCa proteins across eukaryotic lineages. We retrieved the sequences of 65 metazoan NPC and NPCa proteins and searched for their homologs in all eukaryotic phyla for which sequences are available in current databases, such as fungi, green plants, Rhodophytes, Conosa, and Diplomonads (Table 1; Additional data file 1).

Two different phyletic patterns are expected depending on: whether the NPC was a very recent evolutionary innovation and the outcome of independent evolutionary processes in different eukaryotic lineages; or whether it originated before the last eukaryotic common ancestor (LECA [3]). In the first case, very few metazoan NPC and NPCa proteins would have homologs in all eukaryotic lineages; and in the second case, the vast majority of metazoan NPC and NPCa proteins would have homologs in all eukaryotic lineages [26].

Retrieving homologs for NPC and NPCa proteins was unexpectedly difficult, despite the apparent structural conservation of the NPC between fungi and metazoa [8]. The ability to identify and successfully retrieve homologs by BLAST and PSI-BLAST approaches is notably dependent on the evolutionary rates of sequences. For example, attempts to retrieve a rapidly evolving *Arabidopsis thaliana *sequence using a slowly evolving *Homo sapiens *sequence, or *vice versa*, may be unsuccessful if these homologous sequences have evolved beyond recognition. To overcome this limitation, we multiplied the seeds for our BLAST searches. Interestingly, we observed that 40 of the 65 NPC and NPCa proteins studied were present in at least the fungal, animal and plant lineages (Table 1). Furthermore, mining of protist EST databases, notably of stramenopiles, expanded this taxonomical distribution (Table 1), revealing that 48 of the 65 proteins under study were present in bikonts (the grouping of plants and all protists excepted conosa [27]) and in unikonts (the grouping of opisthokonts: metazoa and fungi, and conosa). Among these 48 proteins, 27 of the 33 components of the NPC (Table 1; Figure 1) and 16 of the 17 proteins involved in nucleocytoplasmic transport were conserved in unikonts and bikonts against only four of the 14 proteins associated with the nuclear envelope (Lbr, Narf, Rfbp and Man1; Table 1). Thus, we did not observe any of the outcomes of the two *a priori *models, but we obtained an intermediate picture, in which most but not all of the metazoan NPC and NPCa proteins have homologs in other eukaryotic lineages. A unique and ancient origin of the NPC and, by extension, of the nuclear compartment itself would be favored because similar patterns of distribution would be better explained by an inheritance from the LECA than by multiple convergent recruitments. This claim would be strengthened if phylogenies of these eukaryotic ubiquitous proteins are all in agreement with the eukaryotic tree [26]. Indeed, phylogenetic analyses of these proteins led to trees in which the relationships between the eukaryotic lineages were generally well preserved; most of the trees displaying apparent phylogenetic oddities could be easily rationalized by reconstruction artifacts due to heterogeneity of evolutionary rates (not shown).

Interestingly, the ubiquitous homologs are broadly located on the NPC structure (Figure 1), suggesting that a large fraction of the genes for NPC components originated once, prior to the LECA (27 of the 33 nucleoporins have homologs in unikonts and bikonts), and that the LECA likely had a complex nucleoplasmic transport system (16 of the 17 proteins have homologs in unikonts and bikonts) and possibly a large and modern-type nucleus.

We reckon that one has to be cautious when making conclusions about the lack of homologs in some lineages, such as conosa, for which no complete genome was available when we conducted this study (Table 1; Figure 1). This reduced our ability to shed light on several steps of NPC evolution. In organisms with complete genome sequences available, such as metazoa, fungi, and green plants, an absence may be interpreted as either a true loss, but also as the outcome of evolution beyond recognition. For example, the absence of a metazoan and fungal Nup214/Nup159p homolog in green plants (despite the presence of the homolog of its partner Nup88/Nup82p) may well reflect a true loss of this gene in the green plant lineage or an innovation in the opisthokont lineage (metazoa and fungi). If this absence is proven to be true, it could suggest some limited structural reorganization of the NPC. However, this apparent absence could also simply reflect a fast evolutionary rate for this protein in green plants or in opisthokonts, or both.

Interestingly, eight proteins (Pom121, Gp210, and the lamina-associated proteins Emerin, Otefin, Lamina A/C, Lamina B1 and B2, Lap1 and Lap2) were found only in metazoa, whereas five proteins (Pom152, Pom34, Ndc1, Nup1p and Nup2p) appeared as fungi specific (Table 1). Could this reflect lineage-specific innovations? In metazoa, Pom121 and Gp210 are involved in the anchoring of the NPC to the nuclear membrane [5]. The lack of apparent homologs of these genes in fungi indicates that they likely have an analogous anchoring system. Indeed, structural analyses have shown that three analogous proteins (Pom152, Pom34, and Ndc1) that do not display any sequence similarity with Pom121 and Gp210 perform this function in fungi [6]. These observations favor the hypothesis of a lineage-specific innovation with non-homologous replacement, followed by loss of the ancestral anchoring system in one of the two lineages. Additional information about the NPC anchoring structure in other opisthokonts, and in conosa (for which no homologs of those genes have been detected) may help to determine in which lineage (fungal or metazoan) this replacement occurred. A similar hypothesis could be formulated for the metazoan-specific nucleoporins Nup153 and Nup50. Structural analyses revealed that fungi possess analogues of Nup153 and Nup50 called Nup1p and Nup2p, respectively [5]. As plants harbor a candidate homolog of Nup50, a replacement of these proteins may have occurred specifically in fungi. An alternative explanation would be that they have evolved beyond recognition. Further investigations of structural data, especially from protists and plants, will be required to further test these hypotheses.

### Heterogeneity of evolutionary rates and domain evolution of NPC and NPCa proteins

**Figure 3:**
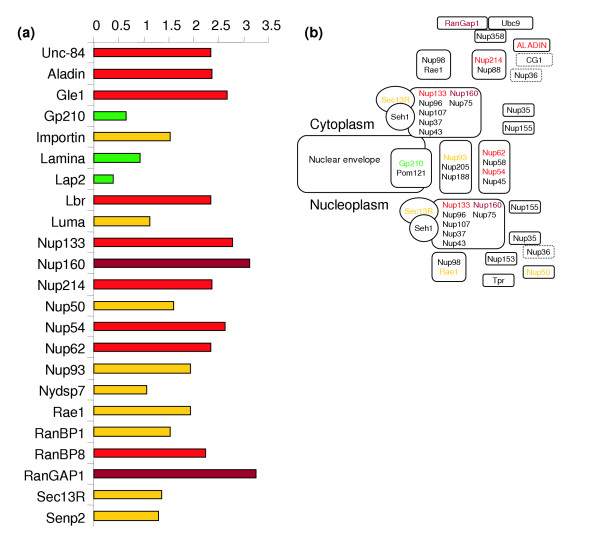
NPC and NPCa protein evloutionary rates. **(a) **Comparison of the evolutionary rates for several NPC and NPCa proteins. The evolutionary rate for a marker corresponds to the average distance estimated between species. **(b) **The evolutionary rates mapped onto the NPC structure with a color code: green, slowly evolving marker (average distance < 1); yellow, marker evolving at an average rate (1 < average distance < 2); red, rapidly evolving marker (2 < average distance < 3); dark red, very rapidly evolving marker (average distance > 3).

**Figure 4:**
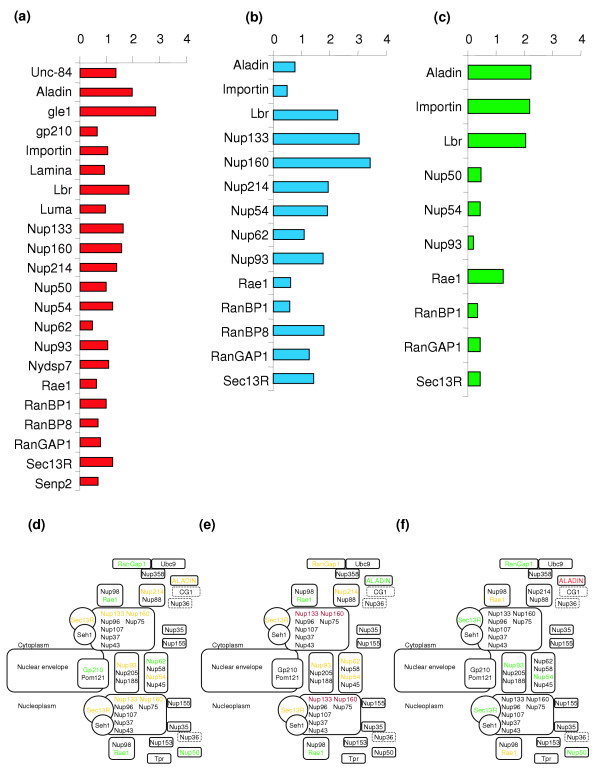
NPC and NPCa protein evloutionary rates within lineages. Comparison of the evolutionary rates of three lineages for several NPC and NPCa proteins, calculated for a marker as the average distance between species of a particular lineage: **(a) **metazoa in red; **(b) **fungi in blue; and **(c) **green plants in green. The evolutionary rate for a marker corresponds to the average distance estimated between species of a given lineage. The evolutionary rates were mapped onto the **(d) **metazoan, **(e) **fungi and **(f) **green plant NPC structures with a color code: green, slowly evolving marker (average distance < 1); yellow, marker evolving at an average rate (1 < average distance < 2); red, rapidly evolving marker (2 < average distance < 3); dark red, very rapidly evolving marker (average distance > 3).

**Figure 5:**
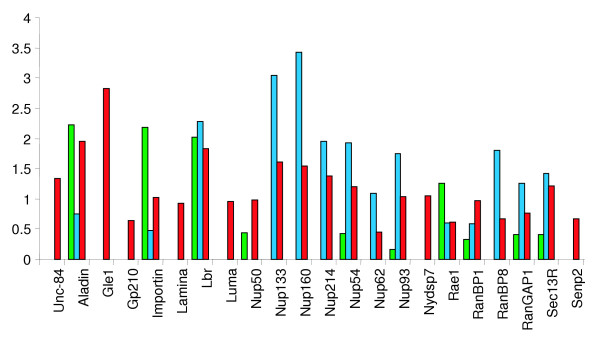
Alternative representation of the evolutionary rates presented in Figure 4a,b,c, allowing a better comparison of the evolutionary rates of several NPC and NPCa proteins between the three lineages (metazoa in red, fungi in blue and green plants in green).

**Figure 6:**
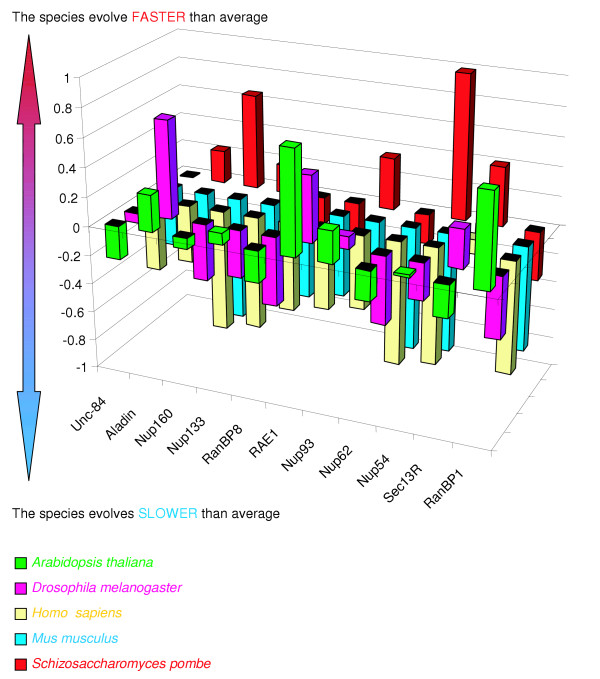
Relative evolutionary rates of several NPC and NPCa proteins for several species (*H. sapiens*, *M. musculus*, *D. melanogaster*, *S. pombe and A. thaliana*), corresponding to the average distance to a given species minus the average distance to any species.

To understand the evolution of NPC protein sequences, we compared evolutionary rates: between markers for all the species (Figure 3); between markers for three given lineages independently (Figures 4 and 5); and within lineages (Figure 6). We produced a very conservative estimate because we considered only the 22 datasets composed of unambiguously aligned sequences having multiple representatives in green plants, fungi, and/or metazoan groups (the datasets used are available in Additional data file 2). Other markers presented too little sequence conservation and/or too limited taxonomic samples in the three lineages analyzed. We show that these 22 ubiquitous proteins present important differences in their rates of evolution (Figure 3a). For instance, some proteins (Nup160 or RanGAP1) displayed on average six times more substitutions than others (Lap2) (Figure 3a). The position within the NPC structure did not explain these differences in evolutionary rates as proteins evolving at either rapid or average rates are uniformly distributed across the NPC and found in almost all of the NPC subcomplexes (Figure 3b). However, such a global average rate of evolution, because it is estimated for all species altogether, is not the most accurate way to describe the evolution of protein sequences, which might be lineage-dependent. Thus, we estimated the evolutionary rates in fungi, metazoa, and plants separately (Figures 4 and 5). This analysis revealed that the markers were not homogeneously slowly or rapidly evolving. In fact, they evolved at different rates in the different lineages, without any general rule and without any obvious correlation with their structural location (Figures 4 and 5). For instance, Nup93 and Nup54 evolved at average rates in metazoa and in fungi, but slowly in plants (Figures 4 and 5). Some markers such as RanGAP1 are slowly evolving in the green plants and in metazoa but evolving at an average rate in fungi, while Importin is slowly evolving in fungi but rapidly evolving in plants and at an average rate in metazoa (Figures 4 and 5). Rae1 protein displays slowly evolving evolutionary rates within fungi and metazoa and average evolving evolutionary rates in plants; Nup133 and Nup160 evolve at average rates within metazoa but very rapidly in fungi, and so on. Evolutionary rates were also sometimes heterogeneous within a given lineage. For instance, Rae1 evolves faster than average in *Drosophila melanogaster *but slower than average in *Mus musculus *and *H. sapiens *(Figure 6).

These irregular rates of evolution, at all levels of analysis (between markers, between lineages and within a lineage) suggest multiple independent adaptations to independent constraints. Because NPC and NPCa proteins are involved in very diverse functions, the contrast between their ubiquitous distribution, their lack of sequence conservation, and their heterogeneity of evolutionary rates probably reflects a higher plasticity of sequences than for NPC structure, which could thus have become frozen very early in eukaryotic evolution.

**Figure 7:**
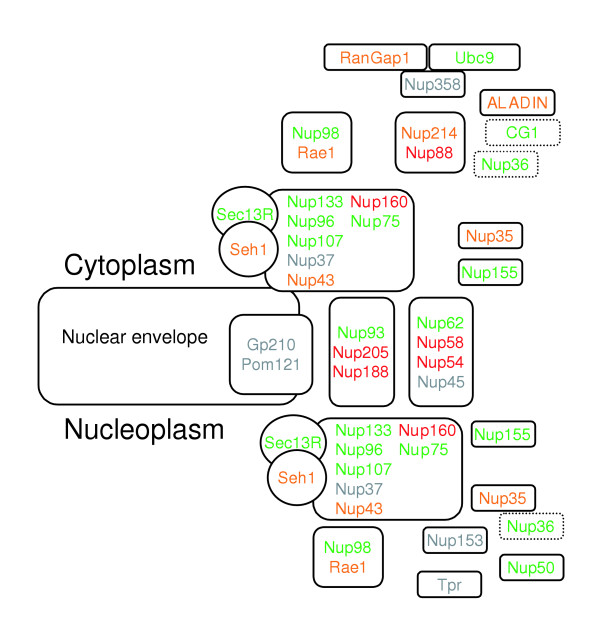
Domain conservation of the proteins constituting the NPC. The color code is: proteins exhibiting the same domain organization in the four species are in green; proteins presenting less than 90% similarity in their organization in domains are in orange; proteins presenting no PFAM domain are in red; proteins for which the structural organization was not studied are in gray.

Yet, if the evolutionary rate of NPC protein sequences is very heterogeneous, the domains detected in 43 proteins by querying the SMART database [28] were generally conserved (Additional data file 10 and Figure 7); 7 out of 43 of the proteins tested presented no domain organization. We found no loss or gain of domains for 23 of the remaining proteins over NPC evolution in four organism representatives of three majors phyla, metazoa, fungi and green plants. Only 12 proteins displayed less than 90% of identical domains between plants, fungi and metazoa, and only half (Narf, Nup214, Luma, Ranbp7, Ranbp8, p30 and Nup35) showed a significant change. For example, Narf has either lost an iron-only hydrogenase domain in *H. sapiens *and *Schizosaccharomyces pombe *or gained it in *D. melanogaster *and *A. thaliana*. Conversely, other proteins (Aladin, Nup43, Rae1, RanGAP1 and Seh1) show variation only in the number of repeated domains. For example, if we take *H. sapiens *as a reference, Aladin seems to have gained two WD domains in *S. pombe*, and one in *D. melanogaster*, and to have lost two such domains in *A. thaliana*.

This strong domain conservation for NPC proteins all over the NPC structure and despite the multiple changes in the rest of the sequence illustrates the strength of the structural constraints acting on NPC and NPCa proteins, probably since LECA.

Thus, while the presence of NPC and NPCa proteins seems to be necessary, most of their sequences can be highly adapted and plastic. These differential evolutionary constraints between sequences and NPC structure are an example of tinkering in eukaryotic evolution, a trick to overcome the frozen structural evolution (that is, the structure and complexes in interaction are preserved, but the sequences of their components vary). Thus, while the global structure of the NPC seems mostly preserved and rigid, it is also strikingly flexible outside the preserved domains, enough to accommodate multiple different functions and to interact with an indefinite number of partners.

### Looking for origins: a possible prokaryotic connection

The age of the NPC structure - as ancient as LECA - raises the question of its origin. The possibility of a pre-LECA NPC deserves consideration. Indeed, a structure comparable to a nucleus (membranes surrounding and isolating the DNA from the rest of the cytoplasm) has been observed in some members of the Planctomycetales, possibly one of the most ancient bacterial phyla [29,30]. However, available data concerning the nature, the composition, the structure, and the function(s) of these nuclear-like structures in Planctomycetales have not yet established whether they were homologous to the eukaryotic nucleus. Importantly, some Methanogens (Archaea) also display intriguing inner membranes [31,32]. Could these structures in prokaryotes and eukaryotes have a common origin or did they appear independently in the three domains of life? Moreover, could viruses have played an important role in the origin of the nucleus and of the NPC as sometimes suggested [33]?

To address this, we tested whether some phylogenetic connections between the eukaryotic NPC components and some putative prokaryote and viral homologs may be proposed. This may provide some answers, even though the absence of a convincing rooting of the Tree of Life does not allow any obvious temporal polarization [34]. For instance, if homologs of the NPC genes were found in prokaryotes, and in particular in Planctomycetales, this could be an argument in favor of a very ancient origin of the genes constituting the NPC (before the separation of the three domains), consistent with a very ancient origin of the nucleus itself. On the other hand, if no prokaryotic homologs are found, the hypothesis of a strictly eukaryotic construction of the NPC (and nucleus) might be most parsimonious.

Hence, we specifically looked for homologous sequences in prokaryotes and viruses, even if they were at first not retrieved when multiple extant eukaryotic seeds were used. Clearly, the large evolutionary distances between eukaryotes and prokaryotes and the heterogeneity of evolutionary rates in sequences complicate such analyses [35,36]. Ancestral sequences inferred using Codeml [37], software taking into account the heterogeneity of rates of evolution, for genes with sufficiently long unambiguously aligned regions provided us with additional seeds. Interestingly, BLAST searches seeded with these ancestral sequences systematically recovered previously identified eukaryotic sequences (a positive control on the quality of ancestral sequences) and sometimes retrieved new prokaryotic sequences that were otherwise undetected (Tables 1 and 2).

**Figure 8:**
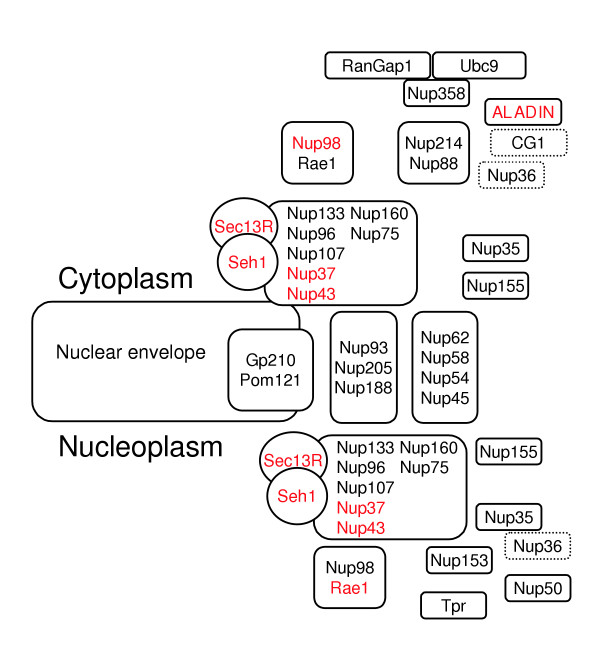
Localization of the six NPC proteins having prokaryotic homologs. The names of those six proteins are in red. All except Aladin are part or associated with the Nup160 subcomplex on the nuclear side.

We found seven proteins with such additional prokaryotic homologs, leading to a total of 15 proteins with prokaryotic homologs: 9 are NPCa proteins (p30, Nurim, Importins, Ha95, Luma, Lbr, Rfbp, Ddx19 and Narf), whereas only 6 are NPC proteins (Nup37, Nup43, Seh1, Rae1, Aladin, Sec13R) (Table 2; Figure 8). All the NPC proteins with prokaryotic homologs detected have WD repeated domains, suggesting that this domain, if not convergent, may be very ancient and would have originated before the separation of the tree domains of life. Five of these NPC proteins are involved in the anchoring system (Ha95, Luma, Nurim, Lbr, Narf). Interestingly, all NPC proteins are localized on the nuclear side, except Aladin, which locates near Nup358 on the cytoplasmic face of NPCs [38]. In addition, two of the NPCa proteins are involved in nucleocytoplasmic transport (Importins and Ddx19). This result is very suggestive because our phylogenetic approach was very conservative: only 31 proteins were used to infer ancestral sequences (see Materials and methods), and the evolutionary distances and the heterogeneity of the evolutionary rates are obviously larger between prokaryotes and eukaryotes than inside eukaryotes alone. This search could then be improved when additional eukaryotic sequences are known.

From these results, an exciting hypothesis may be that an ancient universal system of transmembrane transport was recruited during early eukaryotic evolution (before LECA) to form the NPC. However, too little is currently known about the function of these proteins in prokaryotes to test this hypothesis.

It was interesting to find homologs in Methanosarcinales since those Archaea could display inner membranes. Yet, the absence of undisputable homologs in Planctomycetales, even if the complete genome of *Pirellula *were available, does not support a relationship between their nucleus-like structure and the eukaryotic nucleus. In the detail, the taxonomical distribution of prokaryotic NPC protein homologs is intriguing (Table 2; Additional data files 3, 4, 5, 6, 7). The species harboring these proteins are mainly members of Cyanobacteria for Bacteria and Methanosarcinales for Archaea. The prokaryotic homologs of NPCa proteins are more patchily distributed than those of the NPC proteins. They are mainly present in various phyla of Bacteria such as Proteobacteria, Cyanobacteria, Green non sulfur bacteria or the Cytophagales-Flavobacteria-Bacteroides group. This patchy taxonomical distribution could be explained by multiple independent losses, the proteins being kept in some species for different purposes, but also - and more likely - by several independent gene transfers from eukaryotes to prokaryotes. For Ha95, Luma and Nurim, the hypothesis of lateral gene transfers between metazoa and prokaryotes seems the most likely explanation (see for instance the phylogenies of Luma, found only in metazoan and in *Mesorhizobium loti *and of Nurim, found in some Cyanobacteria plus α-Proteobacteria; Additional data files 5 and 6). These examples of transfers from eukaryotes (and sometimes specifically from metazoa) to prokaryotes suggest that NPC and NPCa proteins can be functional in a prokaryotic cellular context even in the absence of a nuclear compartment. In any case, this illustrates the plasticity, flexibility, multitasking and recruitment potential for these NPC/NPCa proteins, already suggested by their highly specific rates of evolution.

## Conclusion

Our study confirms that most of the metazoan proteins constituting the NPC and involved in nuclear transport have homologs in all eukaryotic lineages, as recently pointed out by Mans *et al*. [3]. Only the main partners of the NPC that localize to the inner membrane appear specific to metazoa. As most of the ubiquitous proteins observed in green plants, fungi, animals and protists are located in all the structural subcomplexes of the NPC, we conclude that the majority of the NPC is homologous in all extant eukaryotes. A core of interacting proteins seems to have been preserved for at least 1.5 billion years, their association being at least as ancient as LECA. How and when this NPC structure originated, however, remains unclear.

At present, most nuclear proteins seem to have no identified prokaryotic homologs. This does not mean, however, that these genes are strictly eukaryotic. They might well have prokaryotic homologs that are too distantly related to be recognized, especially if the origin of eukaryotes involved some sort of quantum evolution [27], with an acceleration of the rate of evolution in the branch leading to extant eukaryotes. Indeed, we found distant prokaryotic homologs of several NPC and NPCa proteins. Some of them were likely recruited by lateral gene transfer from eukaryotes, and it will be interesting to understand the way they adapted their function to a prokaryotic environment. Intriguingly, the presence of prokaryotic homologs of NPC components of the nuclear side may imply the existence of a pre-eukaryotic fragment of the nuclear pore structure.

Finally, our study illustrates that even if NPC and NPCa complexes are built from the same proteins, they display two tempos of evolution, one at the structural level, which became mostly frozen early in eukaryotic evolution, and another, very dynamic, one at the sequence level. The poor conservation of their sequences, the varied evolutionary rates observed in various genes and lineages, the recent replacement of the anchoring system in either the fungi or the metazoa, and the evidence for successful lateral gene transfer (LGT) of these genes, bespeak for this dual evolution of the NPC and NPCa components: structurally rigid but very adaptable in their sequences, a likely reason for the success of the nuclear structure.

## Materials and methods

### Construction of the data sets

Homologous sequences of all the identified nucleoporins in vertebrates and in fungi [5,6,39,40] (completed by the list of proteins published in the Nuclear Protein Database [41]), of proteins involved in the NPC anchoring system [5,6,42], and of several important protein partners in and around the nuclear envelope (Table 1) were retrieved from the National Center for Biotechnology Information [43] with the programs BLASTP, TBLASTN, and PSI-BLAST [44,45]. To avoid incorrect assignment for non-homologous sequences containing the phylogenetically weakly discriminant WD domains and FG repeats, we considered as homologous only those sequences with long stretches of sequence homology outside of these regions with repeats. When no homologous sequences were retrieved outside metazoa, additional searches were performed using each new sequence as a seed to complete the retrieval phase and initiate new searches. Homologous proteins were aligned with ClustalW [46] and the alignment was then manually refined with the ED program of the MUST package [47]. Regions of unambiguous alignment were manually selected using the program NET from the MUST package [47]. All the alignments are available upon request from CB or EB.

Eukaryotic EST databases were mined for each gene with a satisfactory phylogenetic alignment. The EST databases we used (Additional data file 8), included more species than in Mans *et al. *[3] because they notably contained stramenopiles. This approach is far from being ideal, however, because the absence of an EST in a given lineage does not mean that these species do not harbor the corresponding homologs in their genome. In addition, many homologs were probably not retrieved because of the limited size of the databases. Indeed, the largest database (diatoms and conosa, a group including *Dictyostelium *and *Entamoebae *species) provide the largest number of hits.

### Phylogenetic analyses

All protein alignments were used to calculate phylogenetic trees by maximum likelihood (ML), maximum parsimony (MP) and Neighbor Joining (NJ) methods with the programs PHYML version 1.0 [48] (JTT+F+Γ model taking into account among-site rate variations), PMBML (JTT+PMB model) [49] and TREE-PUZZLE version 5.1 [50], PAUP version 4.0 beta [51] and MUST [47].

We selected a few proteins for further in-depth phylogenetic analyses by maximum likelihood (PROML; nine user defined categories) [52] when they presented a broad taxonomic distribution and enough unambiguously aligned sites. Bootstrap values were calculated with an exact procedure (100 replicates were generated using SEQBOOT [52], and trees were inferred by an ML method with Γ distribution using PUZZLEBOOT) to estimate the robustness of phylogenetic inference.

### Estimation of rates of evolution

For 22 proteins with a good alignment and a comparably broad dataset, two conservative estimates of the evolutionary distances between species were deduced from distance matrices, calculated using TREE-PUZZLE version 5.1 with a JTT model corrected by a Γ law and eight categories of rates of evolution [50]. First, the average rate of evolution of a given species in reference to the whole dataset shows if a given species X was evolving slower, faster, or at an average rate relative to other species for this gene. This measure allows the identification of rapidly evolving species. Second, the relative rate of evolution compared only with species of the same lineage (when there are at least three) indicates if a given species X was evolving slower, faster, or at an average rate relative to other members of its lineage. This measure provides an insight into the heterogeneity inside a lineage, and allows one to test, for instance, if the acceleration of rates are phylogenetically consistent. These estimates were calculated by Evospeedometer [53].

### Analysis of domain conservation

The presence of domains in the sequences was investigated using the SMART server [28]. This also allows, in addition to the HMMER searches of the SMART database, which is the default option, detection of outlier homologs and homologs of known structures, signal peptides, internal repeats, intrinsic protein disorders, and PFAM domains. All NPC and NPCa proteins present in at least two of the three lineages, metazoa (*H. sapiens*, *D. melanogaster*), fungi (*S. pombe*) and green plants (*A. thaliana*) were investigated.

### Reconstruction of ancestral sequences

Ancestral sequences were reconstructed for 31 proteins. Only regions of proteins with significantly long, contiguous and unambiguously aligned regions (>200 successive positions) were used (Additional data file 9). A maximum likelihood tree for each of these proteins was calculated by PMBML (JTT+PMB model), with user-defined categories. The topology of this tree was provided as an intree to CODEML [37] (WAG model, pre-estimated alpha parameter by TREE-PUZZLE version 5.1 [50], for eight categories of rates of evolution), which infers the ancestral sequences for each node of the tree. Ancestral sequences were extracted from the outfile of CODEML using ancestRetrieve [54].

## Additional data files

The following additional data are available with the online version of this paper. Additional data file 1 is a table contrasting our phylogenetic-ancestral reconstruction results with those (BLAST-COG based) published in [3]. Additional data file 2 is a zip file containing the 22 datasets we used to compare the evolutionary rates between markers for all the species, between markers for three given lineages independently and within lineages. Additional data file 3 is a PDF file showing the ML tree of the Aladin protein (209 sites). The bootstrap proportions are reported only when they are greater than 75%. Additional data file 4 is a PDF file of the ML tree of the Lbr protein (282 sites). The bootstrap proportions are reported only when they are greater than 80%. Additional data file 5 is a PDF file of the ML tree of the Luma protein (349 sites). The bootstrap proportions are reported only when they are greater than 75%. Additional data file 6 is a PDF file of the ML tree of the Nurim protein (228 sites). The bootstrap proportions are reported only when they are greater than 75%. Additional data file 7 is a PDF file of the ML tree of the Ddx19 protein (282 sites). Purple circles indicate bootstrap proportions greater than 90%. Additional data file 8 is a PDF file including the website addresses of the EST under study. Additional data file 9 is a zip file containing the datasets we used to compute ancestral sequences. Additional data file 10 is a table listing the domains present in the NPC and NPCa proteins in the two metazoa *Homo sapiens *and *Drosophila melanogaster*, the fungus *Schizosaccharomyces pombe *and the green plant *Arabidopsis thaliana*.

## Supplementary Material

Additional data file 1 A table contrasting our phylogenetic-ancestral reconstruction results with the ones (BLAST-COG based) published in [3] Click here for file

Additional data file 2 The 22 datasets we used to compare the evolutionary rates between markers for all the species, between markers for three given lineages independently and within lineages Click here for file

Additional data file 3 ML tree of the Aladin protein (209 sites). The bootstrap proportions are reported only when they are greater than 75% Click here for file

Additional data file 4 ML tree of the Lbr protein (282 sites). The bootstrap proportions are reported only when they are greater than 80% Click here for file

Additional data file 5 ML tree of the Luma protein (349 sites). The bootstrap proportions are reported only when they are greater than 75% Click here for file

Additional data file 6 ML tree of the Nurim protein (228 sites). The bootstrap proportions are reported only when they are greater than 75% Click here for file

Additional data file 7 ML tree of the Ddx19 protein (282 sites). Purple circles indicate bootstrap proportions greater than 90% Click here for file

Additional data file 8 Website addresses of the EST under study Click here for file

Additional data file 9 The datasets we used to compute ancestral sequences Click here for file

Additional data file 10 Domains present in the NPC and NPCa proteins in the twometazoa *Homo sapiens *and *Drosophila melanogaster*, the fungus *Schizosaccharomyces pombe *and the green plant *Arabidopsis thaliana* Click here for file

## Figures and Tables

**Table 1:** Distribution of homologs of the metazoan NPC and NPCa proteins across different lineages of eukaryotes and prokaryotes

Localization/Function	Metazoa	Fungi	Microsporidia	Green plants	Rhodophytes	Conosa	Diplomonads	Diatoms	Kineto-plastids	Alveolates	Archaea	Bacteria
**NPC proteins [5,6,39,40]**												
Integral membrane	Gp210 (Pom210)	*Pom152*	*Pom152*									
	POM121	*Pom34*										
		*Ndc1*										
Spokes	Nup93	*** Nic96p	***	***								
	Nup205	*** Nup192p		***		***						
	Nup188	*** Nup188p		***								
Central transporter	Nup62	*** Nsp1p		***		***						
	Nup58^a^	** Nup49p		**								
	Nup54	*** Nup57p		***								
	Nup45^a^	** Nup49p		**								
Nuclear side	Nup133	*** Nup133p		***								
	Nup96^b^	*** C-nup145p^c^		***								
	Nup107	*** Nup84p		***								
	Nup160	*** Nup120p		***								
	Nup37 [5]	**		**			***				*	***
	Nup43	***		***								***
	Nup75	*** Nup85p	***	***		***				***		
	Seh1 (sec13L)	*** Seh1p	***	***		***			***	***	*	***
	Sec13R	*** Sec13p	***	***	***	***	*	*	**	***	*	**
Cytoplasmic fibrils	Nup35 (MP-44)	*** Nup59p Nup53p		***			***					
	Nup214 (Cain) (Can)	*** Nup159p										
	Nup88	*** Nup82p		***		***				***		
	Ran-Gap1	***	***	***		*			*	**		
	Nup358 (Ranbp2) (Rbp2)	**		**		*						
	Ubc9 (Ube2I)	*** Ubc9p	***	***		***	***		***	***		
Nucleoplamic fibrils (basket)	Nup98	*** N-Nup145p^c ^Nup116p Nup100p^d^		***	**	***						
	Rae1 (gle2)	*** Gle2p	***	***	***	***		***	***	***	*	***
	Tpr	*** Mlp1p Mlp2p		***								
	Nup153	*Nup1p*										
	Nup50 (Npap60L)	*Nup2p*		***								
Other	Nup36^d^	*** Nup100p^d^	***									
	Cg1 (Nlp1)	*** Nup42p (Rip1p)		***								
	Nup155	*** Nup170p Nup157p	***	***	***		***	***		***		
	Aladin [5]	***		***	***	***						***
**NPCa proteins**												
Nuclear periphery [5]	p30			***								***
SUMO-1 protease [55,56]	Senp2	***		***					***	***		
Nuclear mRNA export factor [57]	Tap	***				***						
Nuclear export [58]	Rcc1	***			***	***		*	*			
Nuclear Import	Importin(s)	***		***	***	***		***	***	***	***	***
Nuclear mRNA export [59]	Ddx19 Dbp5	*** Dbp5	***	***	***	***		*	*	***		***
Nuclear mRNA export [60]	Gle1	*** Gle1			***			***				
Nuclear export [10]	Ranbp1	***	***	***	***	***		*	***	***		
Nuclear import Importin 7 [61]	Ranbp7	***		***	***	***				***		
Nuclear import Importin 8 [61]	Ranbp8	***		***	***	***				***		
[62]	Mad1 (Mad1L) (Mad1a)	*** Mad1		***	*							
[62]	Mad2 (Mad2L1) (Mad2a)	*** Mad2		***	***	***	***	***		***		
Nuclear export [10]	Crm1	***		***								
Nuclear mRNA export [63]	HnRNPF			**								
Nuclear mRNA export [63]	HnRNPH			**								
Nuclear mRNA export [63]	HnRNPM	***		***						***		
Nuclear export [58,64]	Ran	***	***	***	***	***	***	***	***	***		
Homolog of unc-84 in *C. elegans *[42]	Unc-84B	***		***			***			***		
Inner nuclear membrane protein [65]	Ha95											**
Inner nuclear membrane protein [42]	Luma											***
Inner nuclear membrane protein [66]	Emerin											
Inner nuclear membrane protein [42,67]	Nurim											***
Inner nuclear membrane protein [42,65]	Man1				*	*		***	*	*		
Lamin B receptor [65]	Lbr	***			***	***		*	*			***
Peripheral protein of the inner nuclear membrane [68]	Otefin											
Ring finger binding protein [65]	Rfbp				*	***	***		***	***		**
Lamina [65]	LaminaA/C	***										
Lamina [65]	LaminaB1											
Lamina [65]	LaminaB2											
Protein co-localized with the nuclear lamina [69]	Narf				***	***	***	*	*	***		***
Lamina associated polypeptid [65,70]	Lap1											
Lamina associated polypeptide [65,71]	Lap2											

^a^Nup58 and Nup45 proteins are generated by alternative splicing of the nup58/nup45 gene mRNA. ^b^Nup96 and Nup98 are cleaved from a 186 kDa precursor protein. ^c^N-Nup145p and C-Nup145p are cleaved from the Nup145p precursor protein. ^d^Nup36 showed 96.8% identity with the carboxy-terminal region of Nup100p. ***, indicates proteins for which the homology with metazoan proteins seems indisputable and allows good alignments; **, indicates proteins with a likely homology; *, indicates proteins for which a putative homology has been detected by BLAST, but for which no alignment was possible; *italic font *corresponds to proteins for which no sequence homology was detected but for which structural analyses revealed similar positions within the nuclear pore complex (NPC); underlined font indicates sequences identified using the reconstruction of ancestral sequences.

**Table 2:** Taxonomic affiliation of NPC and NPCa prokaryotic homologous proteins

Protein name	Archaea	Bacteria	Virus
Ddx19	**Euryarchaeota/Crenarchaeota**	**Proteobacteria, Gram positives, Green non sulfur bacteria...**	
Importin	*Methanosarcina barkeri*	*Anabaena*	
Lbr		*Coxiella burnetii *(gamma-Proetobacteria), *Parachlamydia *sp. (Chlamydiales)	
Ha95		Nostoc fragment	
Luma		*Mezorhizobium loti*	
Aladin	***Methanosarcina acetivorans*** ***Methanosarcina barkeri***	Cyanobacteria mainly (some Planctomycetales and Proteobacteria)	
Nurim		**α-Proteobacteria mainly (some Cyanobacteria and Gram positives)**	
Narf		**Firmicutes, Proteobacteria, CFB group, Green-non sulfur Bacteria**	
Nup37	***Methanosarcina acetivorans***	**Cyanobacteria**	
Nup43		**Cyanobacteria**	
P30		**Proteobacteria**	
		**Cyanobacteria**	
Rae1	***Methanosarcina acetivorans***	**Cyanobacteria**	
Rfbp	*Methanosarcina barkeri, Methanosarcina acetivorans*	Short poorly conserved fragments	
Sec13R	*Methanosarcina acetivorans*	Cyanobacteria	
Seh1	***Methanosarcina acetivorans***	**Cyanobacteria**	

Homologs detected by BLASTP are in bold, whereas homologs detected using ancestral sequences as seed for BLASTP searches are indicated with a standard font.
