# Supplementary material for: The two tempos of nuclear pore complex evolution: highly adapting proteins in an ancient frozen structure
Source: Genome Biol. 2005 Sep 30;6(10):R85. doi: 10.1186/gb-2005-6-10-r85 (PMC1257468; doi:10.1186/gb-2005-6-10-r85)
Supplement: Additional data file 1 — A table contrasting our phylogenetic-ancestral reconstruction results with the ones (BLAST-COG based) published in [3] [file gb-2005-6-10-r85-S1.doc]

| Localization | Metazoa | Fungi | Micro-  sporidia | Green  plants | Rhodo-  phytes | Conosa | Diplomonads | Diatoms | Kinetoplastids | Alveolates | Archaea | Bacteria |
| --- | --- | --- | --- | --- | --- | --- | --- | --- | --- | --- | --- | --- |
| NPC ^5,6,36,37^ | **-**  **Gp210** (Pom210) | *=**Pom152* | *Pom*  *152* |  |  |  |  |  |  |  |  |  |
|  | -POM121 | *=**Pom34* |  |  |  |  |  |  |  |  |  |  |
|  |  | Ndc1 |  |  |  |  |  |  |  |  |  |  |
|  | ?Nup93 | =***Nic96p |  | *** |  |  |  |  |  |  |  |  |
|  | ?Nup205 | **=**  *******  **Nup192p** |  | *** |  | *** |  |  |  |  |  |  |
|  | =Nup188 | ***  Nup188p |  | *** |  |  |  |  |  |  |  |  |
|  | =Nup62 | ***?Nsp1p |  | *** |  | *** |  |  |  |  |  |  |
|  | Nup58^a^ | **+Nup49p |  | ** |  |  |  |  |  |  |  |  |
|  | **=**  **Nup54** | *******  **=**  **Nup57p** |  | *** |  |  |  |  |  |  |  |  |
|  | Nup45 ^a^ | **  Nup49p |  | ** |  |  |  |  |  |  |  |  |
|  | =Nup133 | ***  Nup133p |  | *** |  |  |  |  |  |  |  |  |
|  | **=**  **Nup96^b^** | **?**  *******  **C-nup145p^c^** |  | *** |  |  |  |  |  |  |  |  |
|  | ?Nup107 | = *** Nup84p |  | *** |  |  |  |  |  |  |  |  |
|  | **=**  **Nup160** | **=**  ******* Nup120p |  | *** |  |  |  |  |  |  |  |  |
|  | **+**  **Nup37** ^5^ | ** |  | ** |  |  | *** |  |  |  | *♦ | *** |
|  | +Nup43 | *** |  | *** |  |  |  |  |  |  |  | *** |
|  | ?Nup75 | + *** Nup85p | *** | *** |  | *** |  |  |  | *** |  |  |
|  | +Nup155 | +  ***  Nup170p  **Nup157p** | *** | *** | *** |  | *** | *** |  | *** |  |  |
|  | =Nup35 (MP-44) | + *** Nup59p =  *** Nup53p |  | *** |  |  | *** |  |  |  |  |  |
|  | =Seh1 **(sec13L**) | ***  Seh1p | *** | *** |  | *** |  |  | *** | *** | *♦ | *** |
|  | **+**  **Sec13R** | + *** Sec13p | *** | *** | *** | *** | * | * | ** | *** | *♦ | *** |
|  | =Nup98 | **?**  *******  **N-Nup145p^c^**  **=**  *******  **Nup116p**  **=**  *******  **Nup100p^d^** |  | *** | ** | *** |  |  |  |  |  |  |
|  | **+**  **Rae1**  **(gle2)** | **+**  *******  **Gle2p** | *** | *** | *** | *** |  | *** | *** | *** | *♦ | ***♦ |
|  | =Nup214 (Cain)  (Can) | **=**  *******  **Nup159p** |  |  |  |  |  |  |  |  |  |  |
|  | **=**  **Nup88** | **=**  *******  **Nup82p** |  | *** |  | *** |  |  |  | *** |  |  |
|  | +  Nup358  (**Ranbp2**)  (Rbp2) | ** |  | ** |  | * |  |  |  |  |  |  |
|  | **+**  **Ran-Gap1** | *** | *** | *** |  | * |  |  | * | ** |  |  |
|  | Ubc9  (Ube2I) | ***  Ubc9p | *** | *** |  | *** | *** |  | *** | *** |  |  |
|  | **=**  **Nup153** | ***=***  ***Nup1p*** |  |  |  |  |  |  |  |  |  |  |
|  | +Nup50 (Npap60L) | *+**Nup2p* |  | *** |  |  |  |  |  |  |  |  |
|  | =Tpr | ***Mlp1p Mlp2p |  | *** |  |  |  |  |  |  |  |  |
|  | Nup36^d^ | ***  Nup100p^d^ | *** |  |  |  |  |  |  |  |  |  |
|  | Cg1  (Nlp1) | Nup42p (Rip1p) |  | *** |  |  |  |  |  |  |  |  |
|  | **=**  **Aladin** ^5^ | *** |  | *** | *** | ** |  |  |  |  | *** | *** |
| Nuclear periphery ^5^ | p30 |  |  | *** |  |  |  |  |  |  |  | *** |
| Nuclear mRNA export factor ^48^ | Tap | *** |  |  |  | *** |  |  |  |  |  |  |
| SUMO-1 protease ^49,50^ | Senp2 | *** |  | *** |  |  |  |  | *** | *** |  |  |
| Nuclear Export ^51^ | Rcc1 | *** |  |  | *** | *** |  | * | * |  |  |  |
| Nuclear Import | Importin(s) | *** |  | *** | *** | *** |  | *** | *** | *** | *** | *** |
| Nuclear mRNA export ^52^ | Ddx19  Dbp5 | ***  Dbp5 | *** | *** | *** | *** |  | * | * | *** |  | *** |
| Nuclear mRNA export ^53^ | Gle1 | ***  Gle1 |  |  | *** |  |  | *** |  |  |  |  |
| Nuclear Export ^10^ | +Ranbp1 | *** | *** | *** | *** | *** |  | * | *** | *** |  |  |
| Nuclear Import Importin 7^54^ | Ranbp7 | *** |  | *** | *** | *** |  |  |  | *** |  |  |
| Nuclear Import Importin 8^54^ | +Ranbp8 | *** |  | *** | *** | *** |  |  |  | *** |  |  |
| 55 | Mad1  (Mad1L)  (Mad1a) | ***  Mad1 |  | *** | * |  |  |  |  |  |  |  |
| 55 | Mad2  (Mad2L1)  (Mad2a) | =***Mad2 |  | *** | *** | *** | *** | *** |  | *** |  |  |
| Nuclear Export ^10^ | Crm1 | *** |  | *** |  |  |  |  |  |  |  |  |
| Homologue of unc-84 in *C. elegans* ^38^ | Unc-84B | *** |  | *** |  |  | *** |  |  | *** |  |  |
| Inner nuclear membrane protein ^56^ | Ha95 |  |  |  |  |  |  |  |  |  |  | ** |
| Inner nuclear membrane protein ^38^ | Luma |  |  |  |  |  |  |  |  |  |  | *** |
| Inner nuclear membrane protein ^57^ | **=**  **Emerin** |  |  |  |  |  |  |  |  |  |  |  |
| Inner nuclear membrane protein ^38,58^ | =Nurim |  |  |  |  |  |  |  |  |  |  | *** |
| Inner nuclear membrane protein ^38,56^ | =Man1 |  |  |  | * | * |  | *** | * | * |  |  |
| Lamin B receptor ^56^ | Lbr | *** |  |  | *** | *** |  | * | * |  |  | *** |
| Peripheral protein of the inner nuclear membrane ^59^ | Otefin |  |  |  |  |  |  |  |  |  |  |  |
| Ring finger binding protein ^56^ | Rfbp |  |  |  | * | *** | *** |  | *** | *** |  | ** |
| Lamina ^56^ | **+**  **LaminaA/C** | *** |  |  |  |  |  |  |  |  |  |  |
| Lamina ^56^ | **=**  **LaminaB1** |  |  |  |  |  |  |  |  |  |  |  |
| Lamina ^56^ | **=**  **LaminaB2** |  |  |  |  |  |  |  |  |  |  |  |
| Protein co-localized with the nuclear lamina ^60^ | Narf |  |  |  | *** | *** | *** | * | * | *** |  | *** |
| Lamina associated polypeptid ^56,61^ | =Lap1 |  |  |  |  |  |  |  |  |  |  |  |
| Lamina associated polypeptid ^56,62^ | =Lap2 |  |  |  |  |  |  |  |  |  |  |  |
| Nuclear mRNA export ^63^ | HnRNPF |  |  | ** |  |  |  |  |  |  |  |  |
| Nuclear mRNA export ^63^ | HnRNPH |  |  | ** |  |  |  |  |  |  |  |  |
| Nuclear mRNA export ^63^ | HnRNPM | *** |  | *** |  |  |  |  |  | *** |  |  |
| Nuclear Export ^51,64^ | Ran | *** | *** | *** | *** | *** | *** | *** | *** | *** |  |  |

^a^ Nup58 and Nup45 proteins are generated by alternative splicing of the nup58/nup45 gene mRNA

^b^ Nup96 and Nup98 are cleaved from a 186-kD precursor protein

^c^ N-Nup145p and C-Nup145p are cleaved from the Nup145p precursor protein

^d^ Nup36 showed 96.8% identity with the carboxy-terminal region of Nup100p

The names of markers that were investigated by both studies are in bold. “=” is used when a similar taxonomical distribution was proposed in the two studies. “-“ is used when Mans *et al.*‘s approach detected a broader taxonomical distribution whereas “+” is used when our method detected more homologues than did Mans *et al.*. “?”indicates markers for which the reading of Mans *et al.* did not allow us to conclude if our results identified similar or broader taxonomical distributions. A black square in the column for Archaea and Bacteria indicates when Mans *et al.*’s COG-based approaches was able to propose an homologous sequence in these taxonomical group, that our study did not detect. *** indicates proteins for which the homology with metazoan proteins seems indisputable and allows good alignments; ** indicates proteins with a likely homology; * indicates proteins for which a putative homology has been detected by BLAST, but for which no alignment; was possible; italic font corresponds to proteins for which no sequence homology was detected but for which structural analyses revealed similar positions within the NPC.
