# Supplementary figures and images for: The two tempos of nuclear pore complex evolution: highly adapting proteins in an ancient frozen structure
Source: Genome Biol. 2005 Sep 30;6(10):R85. doi: 10.1186/gb-2005-6-10-r85 (PMC1257468; doi:10.1186/gb-2005-6-10-r85)

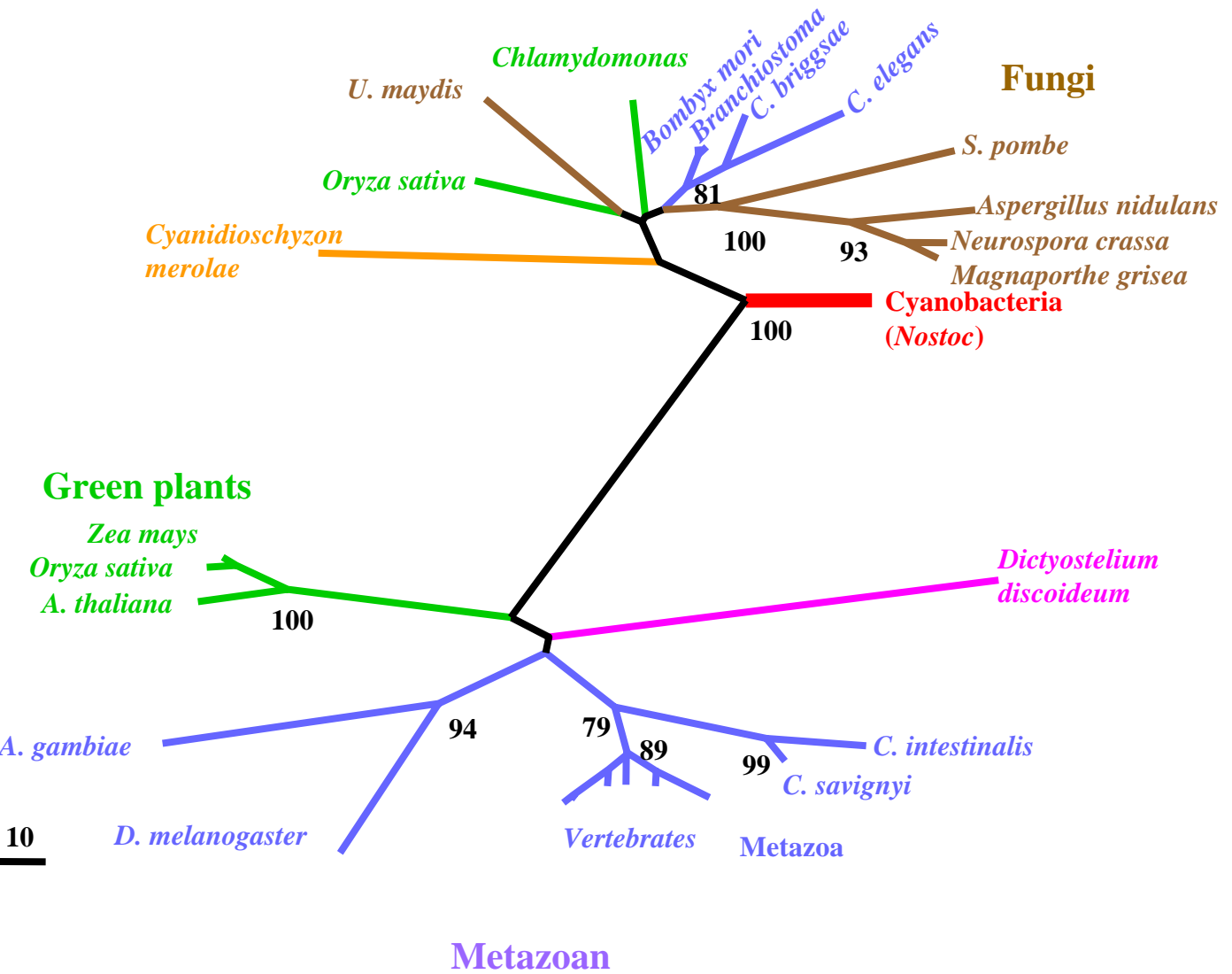

Supplement: Additional data file 3 — ML tree of the Aladin protein (209 sites). The bootstrap proportions are reported only when they are greater than 75% [file gb-2005-6-10-r85-S3.pdf]

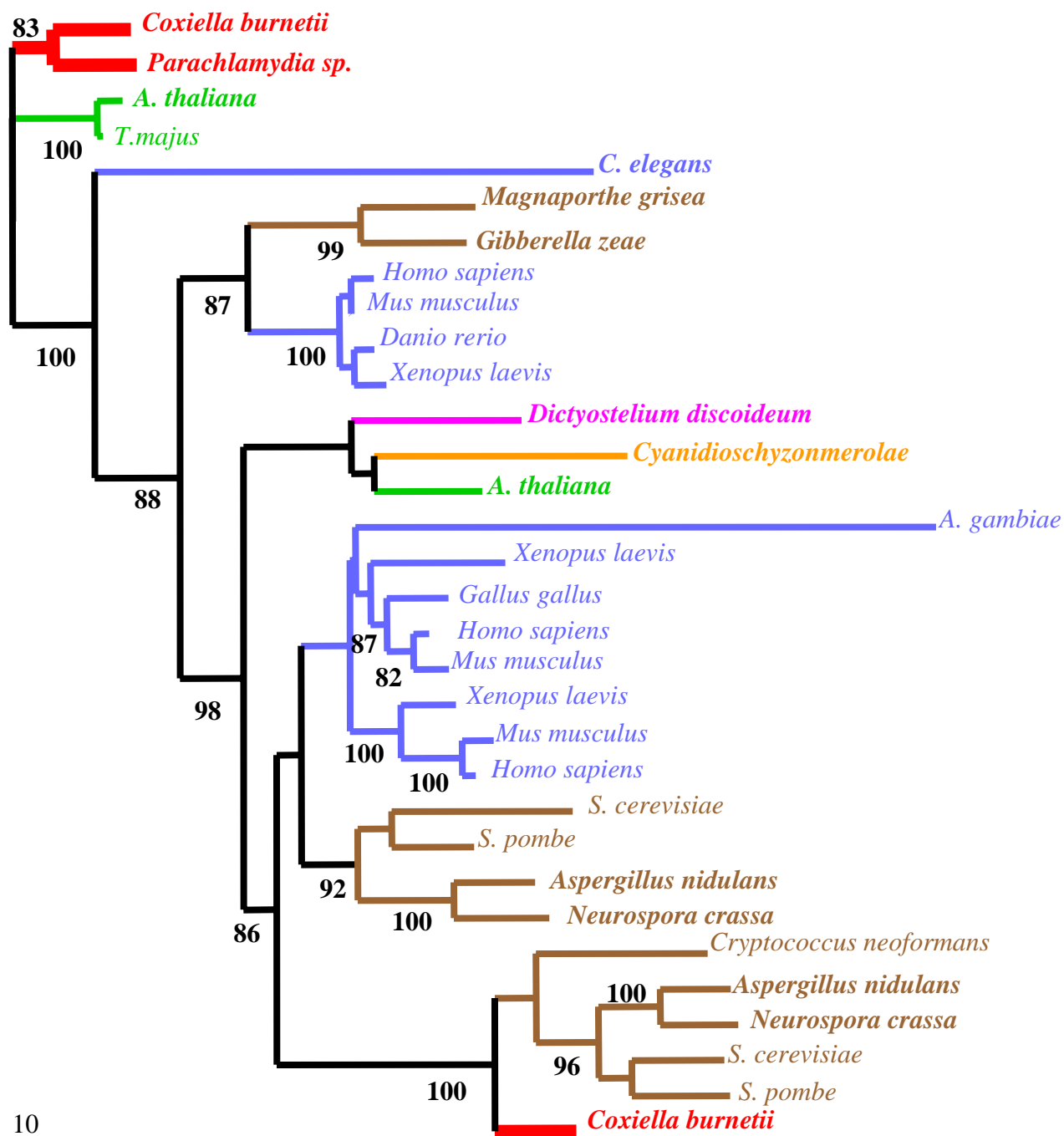

Supplement: Additional data file 4 — ML tree of the Lbr protein (282 sites). The bootstrap proportions are reported only when they are greater than 80% [file gb-2005-6-10-r85-S4.pdf]

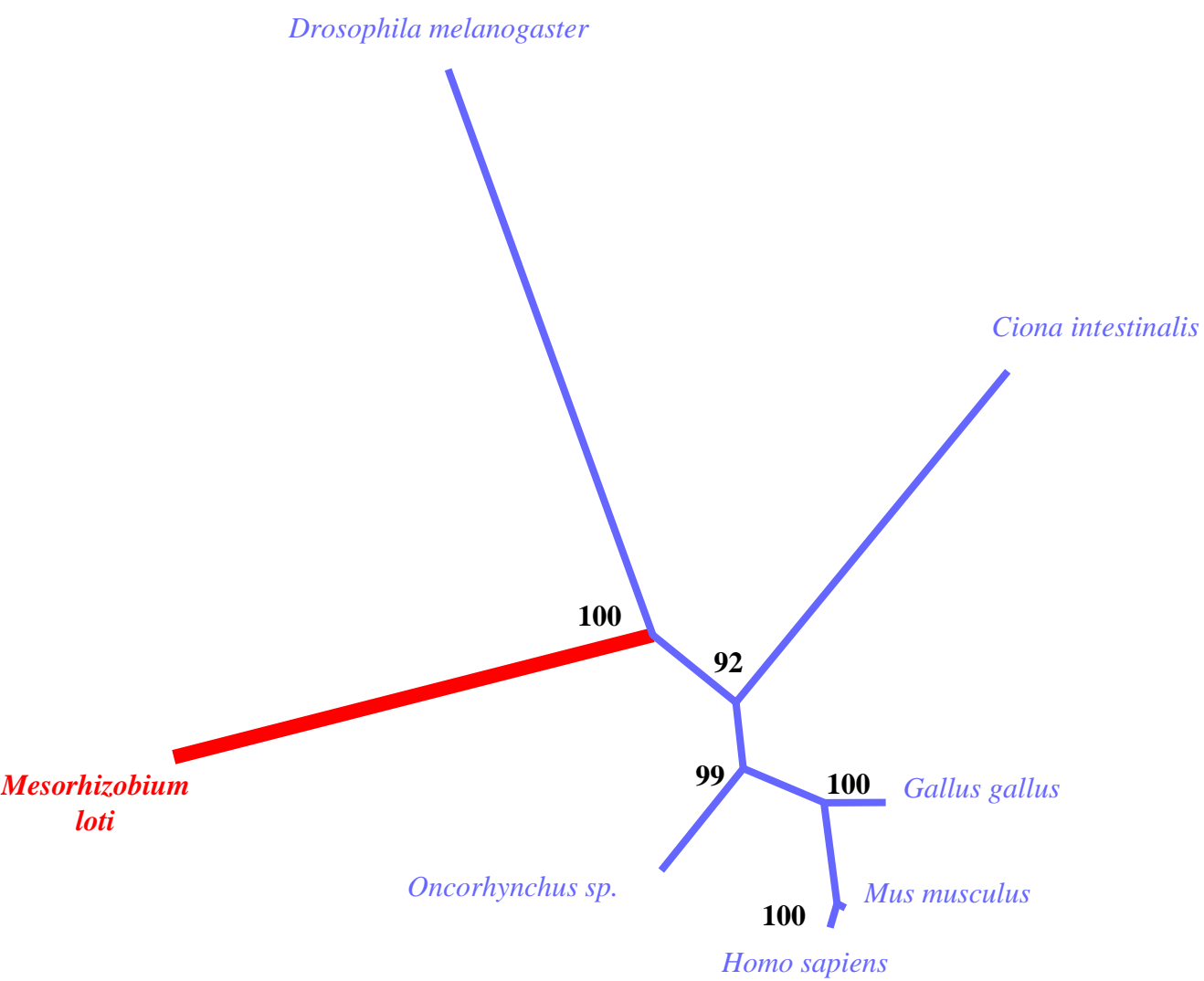

10

Supplement: Additional data file 5 — ML tree of the Luma protein (349 sites). The bootstrap proportions are reported only when they are greater than 75% [file gb-2005-6-10-r85-S5.pdf]

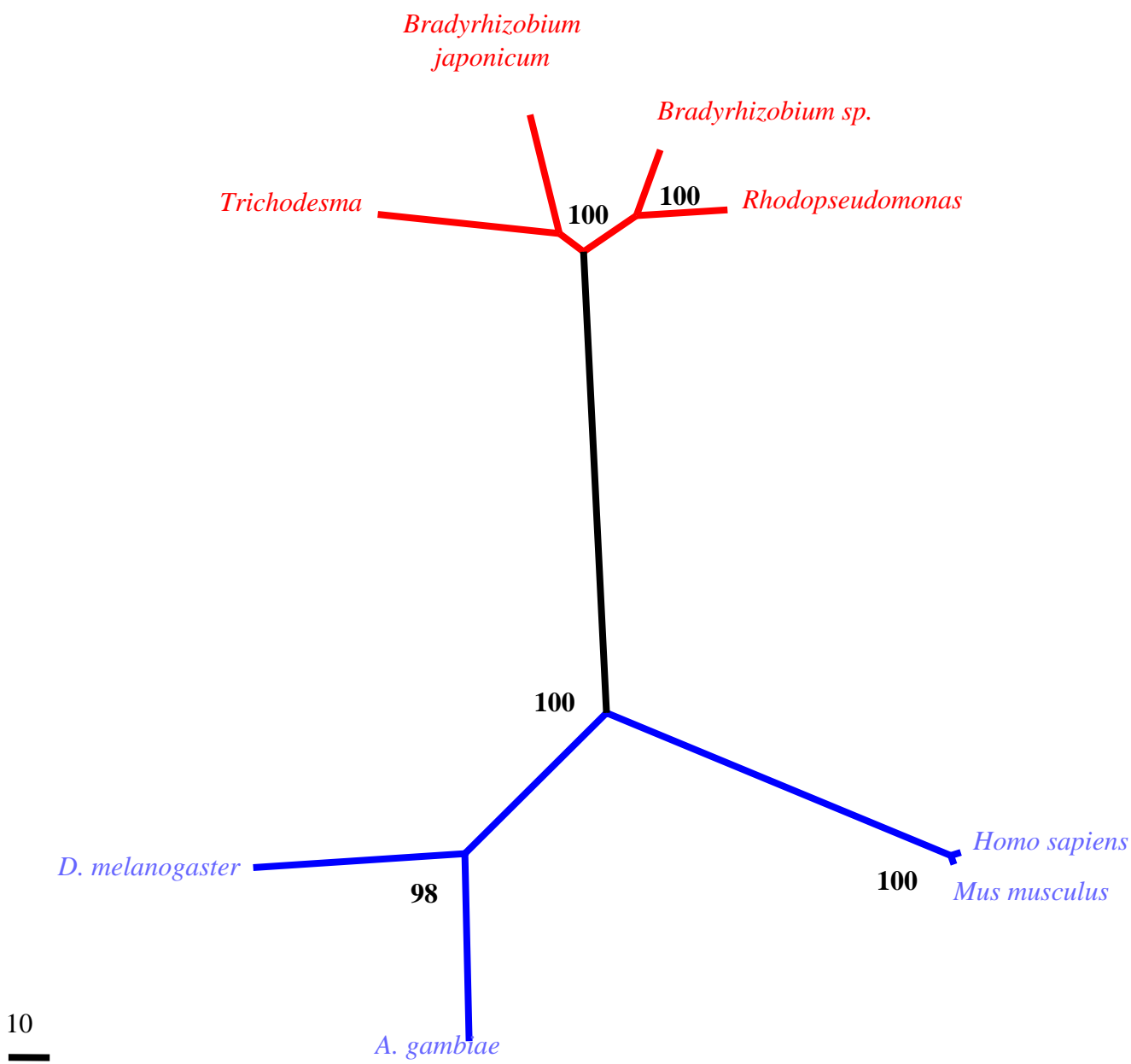

Supplement: Additional data file 6 — ML tree of the Nurim protein (228 sites). The bootstrap proportions are reported only when they are greater than 75% [file gb-2005-6-10-r85-S6.pdf]

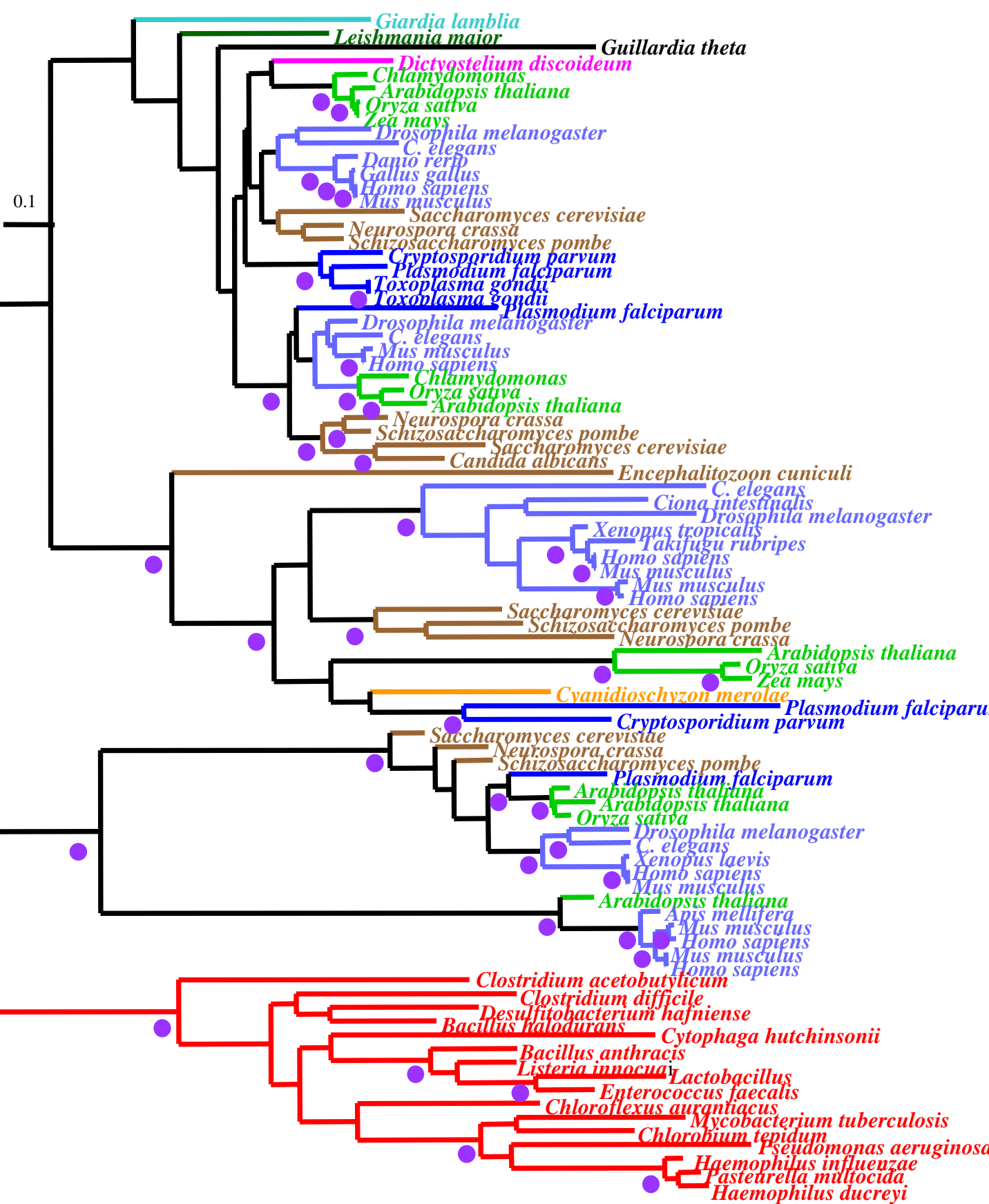

Supplement: Additional data file 7 — ML tree of the Ddx19 protein (282 sites). Purple circles indicate bootstrap proportions greater than 90% [file gb-2005-6-10-r85-S7.pdf]
