# Supplementary material for: The two tempos of nuclear pore complex evolution: highly adapting proteins in an ancient frozen structure
Source: Genome Biol. 2005 Sep 30;6(10):R85. doi: 10.1186/gb-2005-6-10-r85 (PMC1257468; doi:10.1186/gb-2005-6-10-r85)
Supplement: Additional data file 8 — Website addresses of the EST under study [file gb-2005-6-10-r85-S8.pdf]

Metazoa:
D. rerio, S. mansonni, S. japonicum, A. americanum, A. gambiae, B. malayi, C. briggsae: tblastn at ncbi:
Fungi: *P. carinii*, *N. crassa, A. fumigatus*, *A. nidulans, A. terreus*: tblastn at ncbi:
Green plants:
C. reinhardtii: tblastn at ncbi
Rhodophyta:
P. yezoensis: tblastn at http://www.kazusa.or.jp/en/plant/porphyra/EST/
Conosa:
D. dictyostelium: tblastn at ncbi and at http://genome.imb-jena.de/dictyostelium/
E. histolytica, E. invadens, E. terrapinae, E. dispar, *E. moshkovskii*: tblastn at ncbi and http://www.sanger.ac.uk/Projects/Protozoa/
Diatoms/stramenopiles:
***T. pseudonana* at http://genome.jgi-psf.org/cgi-bin/runBlast?db=thaps1**
P. infestans: tblastn at ncbi
Kinetoplastids:
T.cruzi: tblastn at ncbi
T. brucei: tblastn at ncbi and at http://www.sanger.ac.uk/Projects/Protozoa/
L. major: tblastn at ncbi and at http://www.sanger.ac.uk/Projects/Protozoa/
Alveolates:
P. chabaudi: http://www.sanger.ac.uk/Projects/Protozoa/
P. berghei: http://www.sanger.ac.uk/Projects/Protozoa/
***P. vivax*: http://www.sanger.ac.uk/Projects/Protozoa/**
P. yoelii: tblastn at ncbi
T. gondii: tblastn at ncbi and at http://www.sanger.ac.uk/Projects/Protozoa/
T. annulata at http://www.sanger.ac.uk/Projects/Protozoa/
C. parvum: tblastn at ncbi.
E. tennella: http://www.sanger.ac.uk/Projects/Protozoa/

Many thanks at : http://www.sanger.ac.uk/Projects/Protozoa/, http://genome.jgi-psf.org, http://genome.imb-jena.de and http://www.kazusa.or.jp.
