# Supplementary material for: The two tempos of nuclear pore complex evolution: highly adapting proteins in an ancient frozen structure
Source: Genome Biol. 2005 Sep 30;6(10):R85. doi: 10.1186/gb-2005-6-10-r85 (PMC1257468; doi:10.1186/gb-2005-6-10-r85)
Supplement: Additional data file 10 — Domains present in the NPC and NPCa proteins in the twometazoa Homo sapiens and Drosophila melanogaster, the fungus Schizosaccharomyces pombe and the green plant Arabidopsis thaliana [file gb-2005-6-10-r85-S10.doc]

**Table 2**

| **Domains present in the NPC and NPCa proteins in the two metazoa *Homo sapiens* and *Drosophila melanogaster*, the fungus *Schizosaccharomyces pombe* and the green plant *Arabidopsis thaliana*** | | | | | | | |
| --- | --- | --- | --- | --- | --- | --- | --- |
| Protein name | Domain/Motif | *Homo sapiens* | *Drosophila melanogaster* | | *Schizosaccharomyces pombe* | | *Arabidopsis thaliana* |
| *Aladin* |  | 546 aa | 466 aa | | 380 aa | | 447 aa |
|  | WD40 |  |  | | 43-82 | |  |
|  | WD40 | 136-179 | 122-165 | | 85-124 | |  |
|  | WD40 | 181-221 | 169-209 | | 127-166 | | 134-175 |
|  | WD40 | 232-273 | 221-260 | | 169-208 | | 200-241 |
|  | WD40 | 278-315 | 265-301 | | 211-251 | | 246-282 |
|  | WD40 |  | 371-409 | | 254-321 | | 342-386 |
| **CG1** |  | 423 aa | 398 aa | | 475 aa | | 343 aa |
|  | ZF-CCCH | 1-24 | 1-24 | | 1-24 | | 1-26 |
| **CRM1** |  | 1071 aa | 1060 aa | | 1078 aa | | 1076 aa |
|  | IBN_N | 46-112 | 40-106 | | 34-100 | | 37-103 |
| **Ddx19** |  | 448 aa | 460 aa | | 503 aa | | 496 aa |
|  | DEXDc | 50-279 | 92-291 | | 133-329 | | 111-316 |
|  | HELICc | 316-403 | 328-415 | | 366-453 | | 354-442 |
| Emerin | Not done (metazoan sequences only) | | | | | | |
| Gle1 |  | 698 aa | 677 aa | | 480 aa | |  |
| GP210 | Not done (metazoan sequences only) | | | | | | |
| Ha95 | Not done (metazoan sequences only) | | | | | | |
| HnRNPF | Not done (metazoan sequences only) | | | | | | |
| HnRNPF | Not done (metazoan sequences only) | | | | | | |
| HnRNPM | Not done (multiple paralogs) | | | | | | |
| **Importin** |  | 536 aa | 543 aa | 539 aa | | 538 aa | |
|  | IBB | 5-94 | 1-117 | 2-100 | | 4-102 | |
|  | ARM | 112-154 | 126-167 | 109-149 | | 111-152 | |
|  | ARM | 156-196 | 169-209 | 151-191 | | 154-194 | |
|  | ARM | 197-239 | 211-252 | 193-235 | | 195-237 | |
|  | ARM | 242-281 | 254-294 | 238-277 | | 239-278 | |
|  | ARM | 293-323 | 296-336 | 279-319 | | 280-320 | |
|  | ARM | 325-365 | 338-378 | 321-361 | | 322-363 | |
|  | ARM | 367-407 | 380-420 | 363-403 | | 365-405 | |
|  | ARM | 410-450 | 423-463 | 408-448 | | 408-448 | |
| Lamina A/C | Not done (metazoan sequences, multiple paralogs only) | | | | | | |
| Lamina B1 | Not done (metazoan sequences only) | | | | | | |
| Lamina B2 | Not done (metazoan sequences only) | | | | | | |
| Lap1 | Not done (metazoan sequences only) | | | | | | |
| Lap2 |  | 454 aa |  |  | |  | |
|  | LEM | 110-153 |  |  | |  | |
|  | Transmembrane | 411-430 |  |  | |  | |
| **LBR** |  | 475 aa |  | 424 aa | | 369 aa | |
|  | ERG4_ERG24 | 31-475 |  | 3-424 | | 4-396 | |
| *Luma* |  | 400 aa | 376 aa |  | |  | |
|  | Transmembrane | 32-51 | 10-32 |  | |  | |
|  | Transmembrane | 309-331 | 286-308 |  | |  | |
|  | Transmembrane | 346-368 | 323-345 |  | |  | |
|  | Transmembrane | 373-390 |  |  | |  | |
| **Mad1** |  | 718 aa | 730 aa | 689 aa | | 726 aa | |
|  | MAD | 2-716 | 2-726 | 5-689 | | 14-724 | |
| **Mad2** |  | 205 aa | 207 aa | 203 aa | | 209 aa | |
|  | HORMA | 12-201 | 12-205 | 11-201 | | 13-202 | |
| **Man1** |  | 524 aa | 594 aa | 604 aa | | 527 aa | |
|  | RRM | 154-226 | 238-310 | 241-313 | | 169-241 | |
|  | RRM | 251-324 | 336-409 | 341-413 | | 266-340 | |
|  | RRM | 419-498 | 485-564 | 508-587 | | 436-514 | |
| *Narf* |  | 476 aa | 477 aa | 538 aa | | 474 aa | |
|  | Fe_hyd_lg-C | 94-406 | 101-406 | 87-464 | | 81-401 | |
|  | Fe_hyd_SSU |  | 411-466 |  | | 407-464 | |
| **Nup107** |  | 925 aa | 845 aa | 780 aa | | 1077 aa | |
|  | Nup84_Nup100 | 204-910 | 116-830 | 84-769 | | 183-1019 | |
| **Nup133** |  | 1156 aa | 1200 aa | 1162 aa | | 1234 aa | |
|  | Nup133 | 569-1036 | 611-1079 | 481-1008 | | 625-1088 | |
| Nup153 | Not done (metazoan sequences only) | | | | | | |
| **Nup155** |  | 1391 aa | 1365 aa | 1315 aa | | 1475 aa | |
|  | Nucleoporin | 13-1384 | 1-1357 | 9-1308 | | 13-1440 | |
| Nup160 |  | 1436 aa | 1411 aa | 1136 aa | | 1459 aa | |
| Nup188 |  | 1745 aa | 1822 aa | 1564 aa | | 561 aa | |
| Nup205 |  | 1844 aa | 2090 aa | 1683 aa | | 1808 aa | |
| *Nup214* |  | 2119 aa | 1711 aa | 1325 aa | |  | |
|  | WD | 167-207 |  |  | |  | |
|  | WD | 211-249 |  |  | |  | |
| *Nup35* |  | 326 aa | 331 aa |  | | 329 aa | |
|  | MPPN | 167-252 | 184-270 |  | | 180-268 | |
| Nup358 | Not done (metazoan sequences only) | | | | | | |
| **Nup36** |  | 1638 aa | 1961 aa | 1778 aa | | 1076 aa | |
|  | Nucleoporin | 710-882 | 875-1047 | 777-953 | | 905-1075 | |
| Nup37 | Not done (metazoan sequences only) | | | | | | |
| *Nup43* |  | 380 aa | 358 aa | 846 aa | | 358 aa | |
|  | WD40 |  | 2-47 |  | |  | |
|  | WD40 | 60-101 | 64-101 | 80-120 | |  | |
|  | WD40 | 124-157 | 116-155 | 123-163 | | 117-158 | |
|  | WD40 | 160-199 | 159-197 | 166-206 | | 164-203 | |
|  | WD40 | 206-246 | 208-248 | 210-249 | | 212-253 | |
|  | WD40 | 250-290 | 252-292 | 254-300 | |  | |
|  | WD40 |  | 318-355 | 304-342 | |  | |
| Nup45 | Not done (metazoan sequences only) | | | | | | |
| **Nup50** |  | 468 aa | 564 aa |  | | 443 aa | |
|  | RanBP1 | 342-465 | 442-563 |  | | 305-439 | |
| Nup54 |  | 507 aa | 610 aa | 403 aa | | 377 aa | |
| Nup58 |  | 599 aa | 546 aa | 1460 aa | |  | |
| **Nup62** |  | 522 aa | 394 aa | 598 aa | | 739 aa | |
|  | Nsp1_C | 307-429 | 189-305- | 397-520 | | 514-632 | |
| **Nup75** |  | 656 aa | 668 aa | 675 aa | | 716 aa | |
|  | Nucleoporin | 54-605 | 60-609 | 65-640 | | 100-676 | |
| Nup88 |  | 741 aa | 702 aa | 888 aa | | 810 aa | |
| **Nup93** |  | 819 aa | 796 aa | 851 aa | | 861 aa | |
|  | NIC | 231-505 | 220-478 | 235-518 | | 252-550 | |
| **Nup96/Nup98** |  | 1729 aa | 1961 aa | 1778 aa | | 1041 aa | |
|  | Nucleoporin | 727-899 | 875-1047 | 777-953 | | 869-1041 | |
| Nurim | Not done (metazoan and bacterial sequences only) | | | | | | |
| Otefin | Not done (metazoan sequences only) | | | | | | |
| *p30* |  | 272 aa |  |  | | 135aa | |
|  | DUF636 | 155-249 |  |  | | 30-124 | |
| Pom121 | Not done (metazoan sequences only) | | | | | | |
| *Rae1* |  | 368 aa | 360 aa | 352 aa | | 349 aa | |
| (Paralog 1) | WD40 | 28-70 | 21-66 | 19-58 | | 11-53 | |
|  | WD40 | 76-114 | 71-110 | 63-102 | | 62-100 | |
|  | WD40 | 117-157 | 112-153 | 104-146 | | 104-142 | |
|  | WD40 | 250-301 | 245-294 | 234-282 | | 145-181 | |
|  | WD40 |  |  |  | | 230-274 | |
| *Rae1* |  | 328 aa | 326 aa | 320 aa | | 340 aa | |
| (Paralog 2) | WD40 | 2-43 | 1-42 | 1-41 | | 5-45 | |
|  | WD40 | 46-83 | 45-82 | 45-82 | | 48-85 | |
|  | WD40 | 88-124 | 84-123 | 84-123 | | 87-126 | |
|  | WD40 | 127-163 | 126-163 | 219-261 | | 134-170 | |
|  | WD40 | 214-262 | 214-262 |  | | 221-269 | |
|  | WD40 | 265-302 |  |  | |  | |
| **Ran** |  | 225 aa | 216 aa | 216 aa | | 221 aa | |
|  | RAB | 17-180 | 16-216 | 11-169 | | 15-173 | |
| **Ranbp1** |  | 202 aa | 314 aa | 215 aa | | 217 aa | |
|  | RanBD | 26-157 | 103-232 | 75-204 | | 30-158 | |
| *Ranbp8/Ranbp7* |  | 1038 aa | 1049 aa | 1029 aa | | 1040 aa | |
|  | IBN_N | 22-101 | 24-103 | 21-97 | | 25-103 | |
|  | CAS_CSE1 |  |  |  | | 563-947 | |
| *Rangap1* |  | 623 aa | 596 aa | 386 aa | | 545 aa | |
|  | LRR | 84-111 | 44-71 | 31-58 | | 213-240 | |
|  | LRR | 147-174 | 107-134 | 121-148 | | 241-268 | |
|  | LRR | 177-204 | 137-164 | 215-242 | | 269-296 | |
|  | LRR | 215-242 | 203-230 | 243-270 | | 297-324 | |
|  | LRR | 243-270 | 231-258 | 273-300 | | 325-352 | |
|  | LRR | 271-299 | 259-286 |  | | 353-380 | |
|  | LRR | 299-326 | 288-315 |  | | 382-409 | |
|  | LRR | 328-355 | 316-343 |  | | 410-437 | |
|  | LRR | 356-383 |  |  | | 439-466 | |
|  | LRR |  |  |  | | 467-494 | |
| RCC1 | Not done (one metazoan and fungal sequences only) | | | | | | |
| Rbfp | Not done (many paralogs) | | | | | | |
| **Sec13r** |  | 322 aa | 386 aa | 297 aa | | 302 aa | |
|  | WD40 | 1-41 | 31-71 | 1-37 | | 2-39 | |
|  | WD40 | 46-87 | 75-116 | 42-83 | | 45-86 | |
|  | WD40 | 92-133 | 122-163 | 88-129 | | 92-133 | |
|  | WD40 | 139-195 | 170-226 | 134-187 | | 139-192 | |
|  | WD40 | 201-244 | 231-274 | 194-237 | | 199-242 | |
|  | WD40 | 254-290 | 283-319 | 245-283 | | 248-287 | |
| *Seh1* |  | 360 aa | 354 aa | 339 aa | | 302 aa | |
|  | WD40 | 1-40 | 1-40 | 3-42 | | 2-39 | |
|  | WD40 | 46-87 | 46-87 | 49-90 | | 45-86 | |
|  | WD40 | 102-143 | 103-144 | 100-141 | | 92-133 | |
|  | WD40 | 152-201 | 207-248 | 204-247 | | 139-192 | |
|  | WD40 | 267-306 | 259-300 | 278-317 | | 199-242 | |
|  | WD40 |  |  |  | | 248-287 | |
| **Senp2** |  | 590 aa | 468 aa | 568 aa | | 489 aa | |
|  | Peptidase_C48 | 396-588 | 274-467 | 377-566 | | 288-487 | |
| Tap | Not done (one fungal and metazoan sequences only) | | | | | | |
| Trp | Not done (metazoan sequences only) | | | | | | |
| **Ubc9** |  | 185 aa | 159 aa | 157 aa | | 160 aa | |
|  | UBCc | 7-146 | 7-157 | 7-157 | | 9-153 | |
| Unc-84 | Not done (many paralogs) | | | | | | |

Names of proteins exhibiting the same domain organization in the four species are in bold. Names of proteins presenting less than 90% similarity in their organization in domains in the four species are in italic. Names of proteins sharing no domain are underlined. The sequence length is also reported in amino acids (aa) and the description of the domains is given below the table, according to PFAM [72]. WD domain: in higher eukaryotes, the WD domain exists as a small multigene family of highly conserved proteins of about 340 amino acid residues. Structurally, the WD domain consists of eight tandem repeats of about 40 residues, each containing a central Trp-Asp motif (this type of repeat is sometimes called a WD-40 repeat). Zf-CCCH: zinc finger domain. Zinc finger domains are thought to be involved in DNA-binding, and exist as different types, depending on the positions of the cysteine residues. Proteins containing zinc finger domains of the C-x8-C-x5-C-x3-H type include zinc finger proteins from eukaryotes involved in cell cycle or growth phase-related regulation. IBB: importin beta binding domain. This family consists of the importin alpha and importin beta binding domain. The domain mediates formation of the importin alpha beta complex; required for classic import of proteins into the nucleus, through the nuclear pore complex and across the nuclear envelope. ARM: Armadillo/beta-catenin-like repeat. Approximatively 40 amino acid repeat. Tandem repeats form super-helix of helices that is proposed to mediate interaction of beta-catenin with its ligands. ERG4_ERG24: Ergosterol biosynthesis ERG4/ERG24 family. These proteins are highly hydrophobic and seem to contain seven or eight transmembrane regions. Chicken lamin B receptor that is thought to anchor the lamina to the inner nuclear membrane belongs to this family. MAD: mitotic arrest deficient protein. The mitotic spindle checkpoint monitors proper attachment of the bipolar spindle to the kinetochores of aligned sister chromatids and causes a cell cycle arrest in prometaphase when failures occur. HORMA: the HORMA (for Hop1p, Rev7p and MAD2) domain has been suggested to recognize chromatin states that result from DNA adducts, double stranded breaks or non-attachment to the spindle and acts as an adaptor that recruits other proteins. MAD2 is a spindle checkpoint protein that prevents progression of the cell cycle upon detection of a defect in mitotic spindle integrity. RRM: the RRM motif is probably diagnostic of an RNA binding protein. RRMs are found in a variety of RNA binding proteins, including various hnRNP proteins, proteins implicated in regulation of alternative splicing, and protein components of snRNPs. The motif also appears in a few single stranded DNA binding proteins. Fe_hyd_lg_C: iron only hydrogenase large subunit, C-terminal domain. Proteins containing this domain may be involved in the mechanism of biological hydrogen activation and contain 4FE-4S clusters. They can use molecular hydrogen for the reduction of a variety of substances. Fe_hyd_SSU: this family represents the small subunit of the Iron only hydrogenases. Nup84p (nup107 in Metazoa) forms a complex with five proteins, including Nup120p (nup160 in metazoa), Nup85p (nup75 in metazoa), Sec13p, and a Sec13p homolog. This Nup84p complex in conjunction with Sec13-type proteins is required for correct nuclear pore biogenesis. Nup133: RNA undergoing nuclear export first encounters the basket of the nuclear pore. Nup133 is a nucleoporin accessible on the basket side of the pore. RanBP1: Ran is an evolutionary conserved member of the Ras superfamily that regulates all receptor-mediated transport between the nucleus and the cytoplasm. All RanBP1 proteins contain an approximately 150 amino acid residue Ran binding domain. Ran BP1 binds directly to RanGTP with high affinity. There are four sites of contact between Ran and the Ran binding domain. Nsp1_C: Nsp1-like C-terminal region. The NSP1-like protein appears to be an essential component of the nuclear pore complex, for example preribosome nuclear export requires the Nup82p-Nup159p-Nsp1p complex. NIC: nucleoporin interacting component. NIC is part of the nuclear pore complex required for protein transport in the nucleus. IBN_N: importin-beta amino-terminal domain. The exchange of macromolecules between the nucleus and cytoplasm takes place through nuclear pore complexes. Active transport of large molecules through these pore complexes require carrier proteins that shuttle between the two components. CAS_CSE1: CAS/CSE protein, carboxyl terminus. In the nucleus, CAS acts as a nuclear transport factor in the importin pathway. The importin pathway mediates the nuclear transport of several proteins that are necessary for mitosis and further progression. CAS is therefore thought to affect the cell cycle through its effect on the nuclear transport of these proteins. LRR_1: leucine rich repeat. LRRs are 20-29 residue sequence motifs present in tandem arrays in a number of proteins with diverse functions, such as hormone receptor interactions, enzyme inhibition, cell adhesion and cellular trafficking. MPPN: the MPPN (mtotic phosphoprotein N' end) family is uncharacterized, although it probably plays a role in the cell cycle because the family includes mitotic phosphoproteins. UBCc: ubiquitin-conjugating enzymes (UBC or E2 enzymes) catalyze the covalent attachment of ubiquitin to target proteins. DUF636: this family of proteins has no known function, but several strongly conserved cysteine residues. DEXDc: members of this family include the DEAD and DEAH box helicases. Helicases are involved in unwinding nucleic acids. The DEAD box helicases are involved in various aspects of RNA metabolism, including nuclear transcription, pre- mRNA splicing, ribosome biogenesis, nucleocytoplasmic transport, translation, RNA decay and organellar gene expression. HELICc: helicase conserved carboxy-terminal domain. This domain family is found in a wide variety of helicases and helicase related proteins. It may be that this is not an autonomously folding unit, but an integral part of the helicase. RAB: Rab proteins constitute a family of small GTPases that serve a regulatoryrole in vesicular membrane traffic.
